# Supplementary material for: Measurement of Chromosomal Arms and FISH Reveal Complex Genome Architecture and Standardized Karyotype of Model Fish, Genus Carassius
Source: Cells. 2021 Sep 7;10(9):2343. doi: 10.3390/cells10092343 (PMC8471844; doi:10.3390/cells10092343)
Supplement: Supplementary file 1 [file cells-10-02343-s001.zip › cells-1345090-supplementary.pdf]

Supplementary Materials: Measurement of Chromosomal Arms and FISH Reveal Complex Genome Architecture and Standardized Karyotype of Model Fish, Genus *Carassius*

Martin Knytl [1]* and Nicola Reinaldo Fornaini [1]

1. Supplementary Results

1.1. Measurement of the q1, q2, p1 and p2 Values

Each chromosome was labelled by working numeric code (Figure S1). Working numeric codes were assigned randomly and made the counting of chromosomes easier and exact. Four parts of each chromosome, long arm 1 ($q1$), long arm 2 ($q2$), short arm 1 ($p1$), short arm 2 ($p2$), were measured (Figure S2) and calculated as q and p arm according formulas given in the Section 2.3.

1.2. R Analysis

Measured long (q) and short arm (p) values of each chromosome from one metaphase were manually written as a numeric vector in R. Vectors of q and p values were inserted into the table (`data.frame()` function in R). Chromosomal length (*length*), difference between q and p arm (d), q/p arm ratio (r) and centromeric index (i) were calculated and added into the same table. We measured chromosomes of ten metaphases of each *Carassius* species, thus we created ten tables per species, each table contained 100 rows (= 100 chromosomes) and seven columns (= q, p, *length*, d, r and i, including one column of working numeric codes of chromosomes). Tables of each species were associated into the *CAU* list (`list()` function in R). The list of tables for *Carassius auratus* was created according following R scripts, in which *CAU* abbreviation means *C. auratus*, and *CaXX1IL_1_1*, *CaXX1IL_1_2* etc. label a specimen. Numeric codes 1–10 situated behind the q, p, *length*, d, r and i values correspond to the group of values of certain specimen:

```
> CAU <- list (CaXX1IL_1_1=data.frame(p1=p1, q1=q1,
+    length1=p1+q1, d1=q1-p1, r1=q1/p1, i1=100/((q1/p1)+1)),
+    CaXX1IL_1_2 = data.frame(p2=p2, q2=q2, length2=p2+q2,
+    d2=q2-p2, r2=q2/p2, i2=100/((q2/p2)+1)),
+    CaXX1IL_1_3 = data.frame(p3=p3, q3=q3, length3=p3+q3,
+    d3=q3-p3, r3=q3/p3, i3=100/((q3/p3)+1)),
+    CaXX1IL_1_4 = data.frame(p4=p4, q4=q4, length4=p4+q4,
+    d4=q4-p4, r4=q4/p4, i4=100/((q4/p4)+1)),
+    CaXX1IL_1_7 = data.frame(p5=p5, q5=q5, length5=p5+q5,
+    d5=q5-p5, r5=q5/p5, i5=100/((q5/p5)+1)),
+    CaXX1IL_1_8 = data.frame(p6=p6, q6=q6, length6=p6+q6,
+    d6=q6-p6, r6=q6/p6, i6=100/((q6/p6)+1)),
+    CaXX1IL_1_6 = data.frame(p7=p7, q7=q7, length7=p7+q7,
+    d7=q7-p7, r7=q7/p7, i7=100/((q7/p7)+1)),
+    CaXX1IL_2_1 = data.frame(p8=p8, q8=q8, length8=p8+q8,
+    d8=q8-p8, r8=q8/p8, i8=100/((q8/p8)+1)),
+    CaXX1IL_2_2 = data.frame(p9=p9, q9=q9, length9=p9+q9,
+    d9=q9-p9, r9=q9/p9, i9=100/((q9/p9)+1)),
+    CaXX1IL_2_3 = data.frame(p10=p10, q10=q10,
+    length10=p10+q10, d10=q10-p10, r10=q10/p10,
+    i10=100/((q10/p10)+1)))
```

All values were sorted descendingly according to i values using `order()` function in R:

```
> CAU$CaXX1IL_1_1 <-
+   CAU$CaXX1IL_1_1 [order(-CAU$CaXX1IL_1_1$i1),]
> CAU$CaXX1IL_1_2 <-
+   CAU$CaXX1IL_1_2 [order(-CAU$CaXX1IL_1_2$i2),]
> CAU$CaXX1IL_1_3 <-
+   CAU$CaXX1IL_1_3 [order(-CAU$CaXX1IL_1_3$i3),]
> CAU$CaXX1IL_1_4 <-
+   CAU$CaXX1IL_1_4 [order(-CAU$CaXX1IL_1_4$i4),]
> CAU$CaXX1IL_1_7 <-
+   CAU$CaXX1IL_1_7 [order(-CAU$CaXX1IL_1_7$i5),]
> CAU$CaXX1IL_1_8 <-
+   CAU$CaXX1IL_1_8 [order(-CAU$CaXX1IL_1_8$i6),]
> CAU$CaXX1IL_1_6 <-
+   CAU$CaXX1IL_1_6 [order(-CAU$CaXX1IL_1_6$i7),]
> CAU$CaXX1IL_2_1 <-
+   CAU$CaXX1IL_2_1 [order(-CAU$CaXX1IL_2_1$i8),]
> CAU$CaXX1IL_2_2 <-
+   CAU$CaXX1IL_2_2 [order(-CAU$CaXX1IL_2_2$i9),]
> CAU$CaXX1IL_2_3 <-
+   CAU$CaXX1IL_2_3 [order(-CAU$CaXX1IL_2_3$i10),]
```

The *length* and *i* values were dissected from each *data.frame* of the *CAU* list, and put as the new separate folders *length* and *i*, respectively, into the *CAU* list:

```
> CAU$length <- data.frame(length1=CAU$CaXX1IL_1_1$length1,
+    length2=CAU$CaXX1IL_1_2$length2,
+    length3=CAU$CaXX1IL_1_3$length3,
+    length4=CAU$CaXX1IL_1_4$length4,
+    length5=CAU$CaXX1IL_1_7$length5,
+    length6=CAU$CaXX1IL_1_8$length6,
+    length7=CAU$CaXX1IL_1_6$length7,
+    length8=CAU$CaXX1IL_2_1$length8,
+    length9=CAU$CaXX1IL_2_2$length9,
+    length10=CAU$CaXX1IL_2_3$length10)

> CAU$i <- data.frame(i1=CAU$CaXX1IL_1_1$i1,
+    i2=CAU$CaXX1IL_1_2$i2, i3=CAU$CaXX1IL_1_3$i3,
+    i4=CAU$CaXX1IL_1_4$i4, i5=CAU$CaXX1IL_1_7$i5,
+    i6=CAU$CaXX1IL_1_8$i6, i7=CAU$CaXX1IL_1_6$i7,
+    i8= CAU$CaXX1IL_2_1$i8, i9= CAU$CaXX1IL_2_2$i9,
+    i10= CAU$CaXX1IL_2_3$i10)
```

The *mean_length* and *mean_i* columns were calculated separately for each row (all values were still ordered decreasingly according to the *i* value for the purpose to calculate chromosomes of the same category) and added into the *length* and *i* folder, respectively, of the *CAU* list using `apply()` function:

```
> CAU$length$mean_length <- apply (CAU$length[,1:10], 1, mean)
> CAU$i$mean_i <- apply (CAU$i[,1:10], 1, mean)
```

The resulted tables with dissected lengths, their mean (Table S1), *i* values and their mean (Table S2) are shown. The columns *mean_length* and *mean_i* were plotted. Dot plot (Figure 1) was generated using `plot()` function. Parameters of dot plot (`par()` function) were designed for creation of three plots within one figure (=three species). Species labels were included using `legend()` function.

```
> par(mfcol=c(3,1), mai=c(0.53,0.55,0.17,0.1), font=3)
> plot(CAU$i$mean_i, CAU$length$mean_length, pch=16,
+   col="yellow3", ylim = c(15, 45), xlim = c(0, 50),
+   xlab = "centromeric index", ylab = "chromosomal length",
+   ann=TRUE, las=1, abline(v=c(0, 12.5, 25, 35.5, 50),
+   col="gray", lty=2))
```

Dissection and identification of 50 haploid chromosomes: fifty vectors (=50 homologous chromosomal pairs = *chromosome*1–50) were generated using `function(){}`. The first vector *chromosome*1 was composed of 20 *i* values—the first and second highest *i* values were dissected from each metaphase of *C. auratus* (=rows 1 and 2 of *i* column in which *i* values were sorted decreasingly) and identified as chromosome 1 (chr1). The second vector *chromosome*2 was composed of 20 *i* values—third and fourth highest *i* values were dissected from each metaphase of *C. auratus* (=rows 3 and 4) and identified as chr2. The third vector *chromosome*3 was composed of fifth and sixth highest *i* values dissected from each metaphase of *C. auratus* (=rows 5 and 6) and identified as chr3. Up to the last 50th vector *chromosome*50 was composed of 99th and 100th highest *i* values (=the lowest *i* values) dissected from each metaphase of *C. auratus* (=rows 99 and 100) and identified as chr50.

```
> Select_chrome <- function(i,j) {
+    chromosome <- c(CAU$CaXX1IL_1_1$i1[i:j],
+                    CAU$CaXX1IL_1_2$i2[i:j],
+                    CAU$CaXX1IL_1_3$i3[i:j],
+                    CAU$CaXX1IL_1_4$i4[i:j],
+                    CAU$CaXX1IL_1_7$i5[i:j],
+                    CAU$CaXX1IL_1_8$i6[i:j],
+                    CAU$CaXX1IL_1_6$i7[i:j],
+                    CAU$CaXX1IL_2_1$i8[i:j],
+                    CAU$CaXX1IL_2_2$i9[i:j],
+                    CAU$CaXX1IL_2_3$i10[i:j])
+ }

> chromosome1 <- Select_chrome(1,2)
> chromosome2 <- Select_chrome(3,4)
> chromosome3 <- Select_chrome(5,6)
> chromosome4 <- Select_chrome(7,8)
> chromosome5 <- Select_chrome(9,10)
> chromosome6 <- Select_chrome(11,12)
> chromosome7 <- Select_chrome(13,14)
> chromosome8 <- Select_chrome(15,16)
> chromosome9 <- Select_chrome(17,18)
> chromosome10 <- Select_chrome(19,20)
> chromosome11 <- Select_chrome(21,22)
> chromosome12 <- Select_chrome(23,24)
> chromosome13 <- Select_chrome(25,26)
> chromosome14 <- Select_chrome(27,28)
> chromosome15 <- Select_chrome(29,30)
> chromosome16 <- Select_chrome(31,32)
> chromosome17 <- Select_chrome(33,34)
> chromosome18 <- Select_chrome(35,36)
> chromosome19 <- Select_chrome(37,38)
> chromosome20 <- Select_chrome(39,40)
> chromosome21 <- Select_chrome(41,42)
```

```
> chromosome22 <- Select_chrome(43,44)
> chromosome23 <- Select_chrome(45,46)
> chromosome24 <- Select_chrome(47,48)
> chromosome25 <- Select_chrome(49,50)
> chromosome26 <- Select_chrome(51,52)
> chromosome27 <- Select_chrome(53,54)
> chromosome28 <- Select_chrome(55,56)
> chromosome29 <- Select_chrome(57,58)
> chromosome30 <- Select_chrome(59,60)
> chromosome31 <- Select_chrome(61,62)
> chromosome32 <- Select_chrome(63,64)
> chromosome33 <- Select_chrome(65,66)
> chromosome34 <- Select_chrome(67,68)
> chromosome35 <- Select_chrome(69,70)
> chromosome36 <- Select_chrome(71,72)
> chromosome37 <- Select_chrome(73,74)
> chromosome38 <- Select_chrome(75,76)
> chromosome39 <- Select_chrome(77,78)
> chromosome40 <- Select_chrome(79,80)
> chromosome41 <- Select_chrome(81,82)
> chromosome42 <- Select_chrome(83,84)
> chromosome43 <- Select_chrome(85,86)
> chromosome44 <- Select_chrome(87,88)
> chromosome45 <- Select_chrome(89,90)
> chromosome46 <- Select_chrome(91,92)
> chromosome47 <- Select_chrome(93,94)
> chromosome48 <- Select_chrome(95,96)
> chromosome49 <- Select_chrome(97,98)
> chromosome50 <- Select_chrome(99,100)
```

Box plot (Figure 2) was generated using `boxplot()` function. Parameters of box plot (`par()` function) were designed for creation of three plots within one figure. The legend was included using `text()` function.

```
> par(mfcol=c(3,1), mai=c(0.53,0.55,0.1,0.1), font = 4)
> boxplot(chromosome1, chromosome2, chromosome3, chromosome4,
+   chromosome5, chromosome6, chromosome7, chromosome8,
+   chromosome9, chromosome10, chromosome11, chromosome12,
+   chromosome13, chromosome14, chromosome15, chromosome16,
+   chromosome17, chromosome18, chromosome19, chromosome20,
+   chromosome21, chromosome22, chromosome23, chromosome24,
+   chromosome25, chromosome26, chromosome27, chromosome28,
+   chromosome29, chromosome30, chromosome31, chromosome32,
+   chromosome33, chromosome34, chromosome35, chromosome36,
+   chromosome37, chromosome38, chromosome39, chromosome40,
+   chromosome41, chromosome42, chromosome43, chromosome44,
+   chromosome45, chromosome46, chromosome47, chromosome48,
+   chromosome49, chromosome50, ylim=c(-1, 50), xlim=c(1, 50),
+   horizontal = FALSE, ylab = "centromeric index",
+   xlab = "chromosome", las = 1, pch = 20, whisklty = 3,
+   boxcol = "yellow3", boxfill = gray(0.95), boxlwd = 2,
+   boxwex = 0.7, names = 1:50)
> text(51, 40, "Carassius auratus", col = "yellow3", adj = 1)
```

R analysis of *C. carassius* and *C. gibelio* was done similarly as described in *C. auratus* above. Scripts of *C. carassius* and *C. gibelio* are available on https://www.github.com upon request. The $q + p$ arm measurements arranged within tables created using the first R command of this supplement can be accessible in the form of RData file provided as R workspace, also upon request.

The following scripts involve all three species. Vectors *chromosome*1–*chromosome*50 were associated into the lists ($CAU\_chrome1\_50$, $CCA\_chrome1\_50$, and $CGI\_chrome1\_50$). The *i* values of each chromosome were separately compared among all species using 50 box plots (Figure 3). Here we show how were parameters of box plots entered and how was the box plot for chr1 with the legend called:

```
> par(mfcol=c(25,2), mar=c(0.1,2,0.1,0.1), cex.axis = 0.7,
+   ann = F, font = 2, las = 1, yaxp = c(0, 50, 10),
+   xaxt = "n")
> boxplot(CAU_chrome1_50$chromosome1,
+   CCA_chrome1_50$chromosome1, CGI_chrome1_50$chromosome1,
+   ylim = c(1, 50), whisklty = 3, boxcol = c('yellow3', 2, 4),
+   boxfill = gray(0.95), boxlwd = 3, boxwex = 0.5)
> text(3.6, 30, "chr1", adj = 1)
```

Similarly other box plots and legend were called for chr2–50. Output values ($*stats*) were called and the *i* values of each chromosome were compared among all species. The following R results show an example of the chr1. There is the script of the first box plot in parentheses followed by result from the console of RStudio. The printed table $*stats* shown below generated values of the minimum (Q_0) on the first row [1,], first quartile (Q_1) on the second row [2,], median (Q_2) on the third row [3,], third quartile (Q_3) on the fourth row [4,], and maximum (Q_4) on the fifth row [5,]. The first [,1], second [,2], and third [,3] columns represent *i* values of *C. auratus*, *C. carassius*, and *C. gibelio*, respectively. We focused on the *i* values within Q_1–Q_3. In this example, *i* values within Q_1–Q_3 range from 47.48232 to 48.74874 in *C. auratus*, from 46.66665 to 48.91326 in *C. carassius*, and from 47.76151 to 49.08830 in *C. gibelio*. All three species share the range of *i* values between 47.76 and 48.74 thus we concluded that chr1 is similar in all three investigated species.

```
> (boxplot(CAU_chrome1_50$chromosome1,
+   CCA_chrome1_50$chromosome1, CGI_chrome1_50$chromosome1,
+   ylim = c(1, 50), whisklty = 3, boxcol = c('yellow3', 2, 4),
+   boxfill = gray(0.95), boxlwd = 3, boxwex = 0.5))

$stats
         [,1]     [,2]     [,3]
[1,] 46.06040 44.88883 46.50152
[2,] 47.48232 46.66665 47.76151
[3,] 47.86878 48.28373 48.55148
[4,] 48.74874 48.91326 49.08830
[5,] 49.40714 49.86277 50.80790

$n
[1] 20 20 20

$conf
         [,1]     [,2]     [,3]
[1,] 47.42136 47.49001 48.08272
[2,] 48.31621 49.07745 49.02023
```

```
$out
[1] 45.13813 44.39824

$group
[1] 3 3

$names
[1] "" "" ""
```

Standardized karyotype was determined based on the Q_2 range of each chromosome. Table with *median* values of each species was generated using `sapply()` function (Table 2). Chromosomal category was assigned by `if(){}` condition, and `for(){}` function was used for loop. Chromosomal categories were added into Table 2. Determination of chromosomal categories is shown for *C. auratus*:

```
> CAU_CCA_CGI_median <- data.frame(CAU_median_i=sapply
+    (CAU_chrome1_50, median), CCA_median_i=sapply
+    (CCA_chrome1_50, median), CGI_median_i=sapply
+    (CGI_chrome1_50, median))

> for(i in 1:50) {
+    if(CAU_CCA_CGI_median$CAU_median[i]>=37.5) {
+        CAU_CCA_CGI_median$CAU_category[i] = "m"
+    }
+    else if (CAU_CCA_CGI_median$CAU_median[i]>=25 &
+        CAU_CCA_CGI_median$CAU_median[i]<37.5){
+        CAU_CCA_CGI_median$CAU_category[i] = "sm"
+    }
+    else if (CAU_CCA_CGI_median$CAU_median[i]>=12.5 &
+        CAU_CCA_CGI_median$CAU_median[i]<25){
+        CAU_CCA_CGI_median$CAU_category[i] = "st"
+    }
+    else if (CAU_CCA_CGI_median$CAU_median[i]>0 &
+        CAU_CCA_CGI_median$CAU_median[i]<12.5) {
+        CAU_CCA_CGI_median$CAU_category[i] = "a"
+    }
+    else {CAU_CCA_CGI_median$CAU_category[i] = "T"
+    }
+ }
```

2. Supplementary Figures and Tables

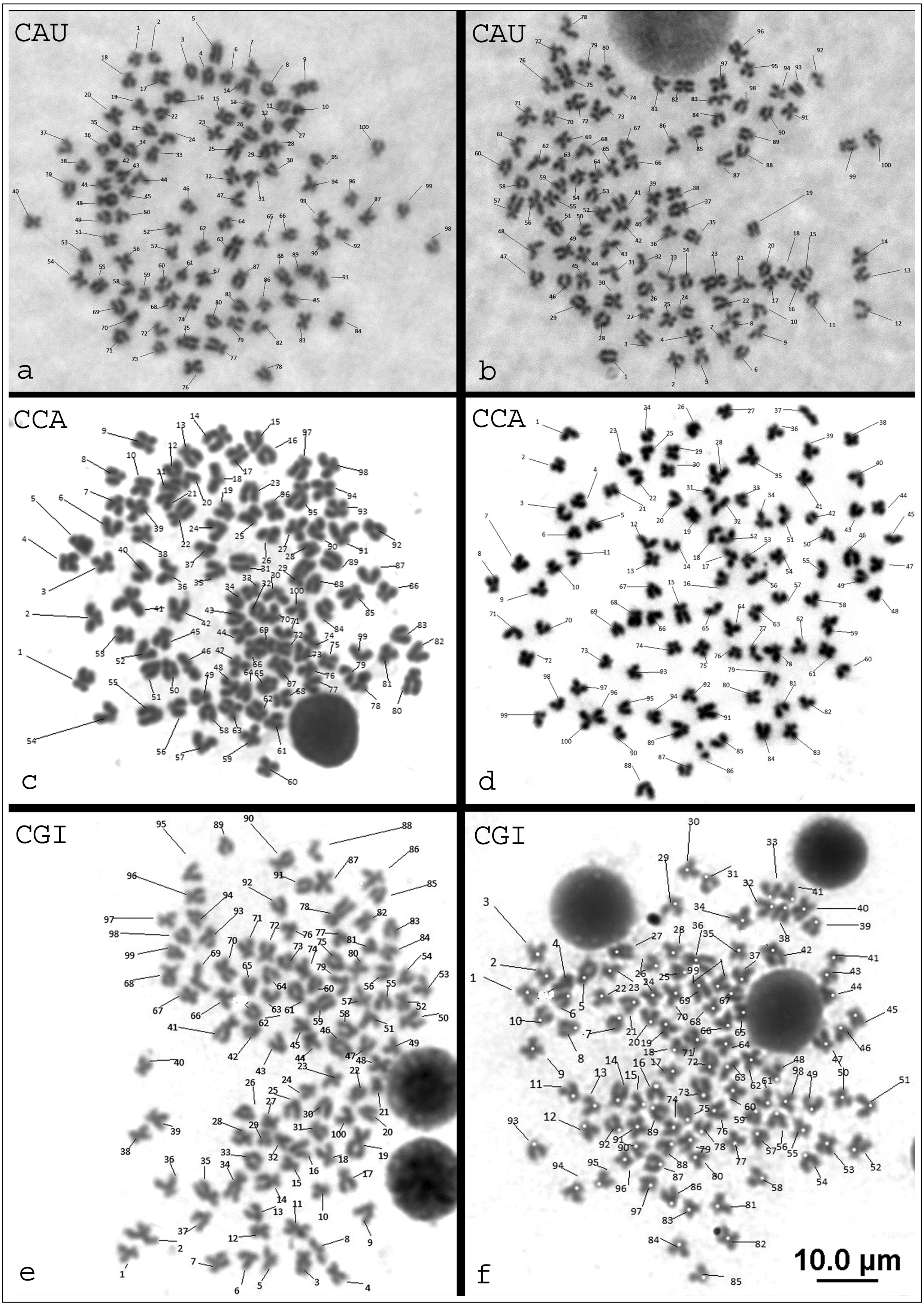

Figure S1. Giemsa-stained chromosome metaphases (B&W) used for long (q) and short (p) chromosome arms measurement. The Figure shows one representative metaphase for each sex of each species. Each chromosome has a working numeric code 1–100. Metaphases show 100 chromosomes in (**a**) *Carassius auratus* (CAU) male, (**b**) *C. auratus* female, (**c**) *C. carassius* (CCA) male, (**d**) *C. carassius* female, (**e**) *C. gibelio* (CGI) male and (**f**) *C. gibelio* female, white circles in (**f**) indicate centromeric regions assigned as the narrowest part of chromosome.

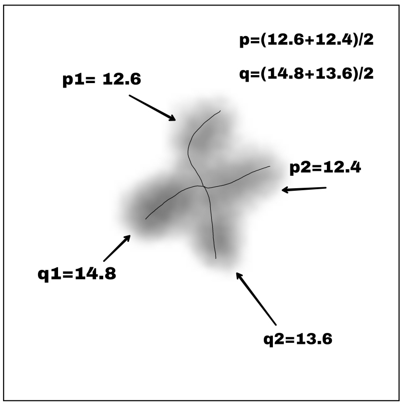

Figure S2. Giemsa-stained chromosome dissected from metaphase. Graphical example of the measurement of metacentric chromosome with four chromosomal arms (two chromatids). Two thin lines which lead through all four arms cross in the centromeric region—the narrowest part of chromosome. Long arm 1 ($q1$), long arm 2 ($q2$), short arm 1 ($p1$), and short arm 2 ($p2$) have 14.8, 13.6, 12.6, and 12.4 pixels, respectively. Calculation of the $q + p$ arm length is shown at the top right of the figure.

Table S1. *Carassius auratus q + p lengths* were dissected from each metaphase and added into separate table. The last column (*mean_length*) shows arithmetic mean of length used for dot plot (Figure 1).

chr	length1	length2	length3	length4	length5	length6	length7	length8	length9	length10	mean_length
1	41.21	40.40	30.25	27.45	29.74	46.99	47.46	35.38	24.49	12.94	33.63
2	42.17	36.95	52.36	40.42	33.10	36.18	46.20	21.82	18.01	21.74	34.89
3	44.76	38.98	36.54	36.66	41.53	39.98	49.60	20.85	21.45	20.49	35.08
4	50.35	38.05	33.25	33.77	34.32	33.20	35.57	18.22	20.32	17.49	31.45
5	39.81	39.35	38.03	34.49	37.36	36.94	26.78	20.90	31.17	21.28	32.61
6	46.71	33.17	38.91	33.50	36.18	30.68	45.60	15.23	15.16	25.11	32.03
7	49.76	42.38	42.81	36.90	39.84	45.20	50.39	20.24	20.49	22.41	37.04
8	49.22	33.66	48.67	43.43	29.62	39.37	49.48	18.19	20.49	23.97	35.61
9	41.18	46.24	32.35	39.87	30.02	34.60	32.24	20.36	23.13	16.45	31.64
10	37.74	32.72	25.50	33.00	36.84	43.22	42.93	19.33	19.19	24.63	31.51
11	45.60	33.91	31.37	39.35	26.47	28.00	41.57	25.21	28.82	26.47	32.68
12	32.90	34.80	39.45	35.42	33.72	35.60	45.18	23.85	20.36	21.47	32.27
13	34.28	41.49	32.12	34.86	45.10	24.14	42.98	16.35	20.90	22.12	31.43
14	34.24	31.92	30.83	29.57	36.83	36.73	31.30	20.54	23.38	18.91	29.42
15	37.72	35.02	28.57	47.30	36.05	39.20	37.21	20.20	13.59	23.30	31.82
16	31.29	34.29	34.64	43.08	37.10	35.87	46.03	17.86	18.20	17.15	31.55
17	39.13	36.27	31.53	38.49	34.93	37.35	36.71	20.16	26.85	20.54	32.20
18	41.30	39.55	28.57	35.01	36.87	38.16	37.82	21.61	15.92	18.21	31.30
19	42.64	39.85	41.80	33.62	52.47	42.11	39.78	20.80	16.17	27.86	35.71
20	41.11	44.78	42.62	33.95	34.43	41.72	36.37	16.96	18.50	19.59	33.00
21	51.88	34.91	30.87	36.76	48.89	36.74	33.17	17.94	22.38	23.86	33.74
22	57.96	56.97	32.68	33.93	45.04	38.89	48.02	14.94	20.12	20.48	36.90
23	43.09	32.08	35.44	47.79	40.05	34.30	51.13	20.91	15.10	24.81	34.47
24	40.73	43.09	34.17	40.11	39.38	38.82	33.03	21.00	19.42	28.28	33.80
25	32.42	41.49	47.51	27.06	51.43	37.02	41.15	20.64	22.79	27.59	34.91
26	38.40	46.74	46.69	42.19	38.49	43.66	31.73	20.06	23.52	14.02	34.55
27	40.35	35.46	28.92	43.39	29.06	40.26	39.06	20.63	34.19	20.92	33.22
28	35.95	39.18	43.12	59.41	37.58	32.28	45.81	16.47	24.30	28.01	36.21
29	37.08	30.13	31.49	36.73	37.81	40.99	34.03	21.27	18.42	17.78	30.57
30	41.29	30.73	35.20	34.27	33.41	31.29	39.52	35.93	19.77	24.34	32.58
31	43.14	48.88	39.23	35.25	45.70	32.52	37.73	22.70	19.15	21.12	34.54
32	36.26	35.73	39.34	38.56	45.00	34.96	28.84	18.84	22.75	24.33	32.46
33	42.10	35.90	42.58	48.79	29.10	37.86	40.87	22.41	21.42	31.30	35.23
34	34.65	48.84	49.13	36.59	32.59	42.16	42.51	19.63	28.22	22.15	35.65
35	37.96	29.52	37.84	38.31	38.67	33.99	66.63	22.03	14.31	23.62	34.29
36	30.33	41.25	50.50	49.62	47.41	38.42	46.99	14.37	23.41	20.75	36.30
37	53.25	32.84	43.27	30.93	30.12	38.41	39.33	27.40	20.71	25.04	34.13
38	48.79	33.37	42.88	31.53	48.05	40.66	47.25	19.98	16.23	25.60	35.43
39	48.63	33.47	33.39	38.64	37.24	32.00	40.49	11.78	21.46	15.38	31.25
40	40.21	30.54	38.15	40.45	40.56	50.84	40.05	19.70	21.17	33.30	35.50
41	28.98	45.12	43.81	48.80	33.37	36.82	36.74	17.47	19.68	15.96	32.68
42	31.50	40.72	37.91	40.83	33.33	42.82	36.16	19.89	23.17	28.38	33.47
43	33.02	30.22	38.27	65.58	45.93	43.16	42.57	22.07	28.44	25.84	37.51
44	33.77	37.39	32.15	55.64	27.95	33.33	16.94	21.62	32.65	26.01	31.74
45	40.19	43.10	44.94	50.72	33.83	40.86	36.79	20.31	17.42	21.81	35.00
46	43.03	34.54	36.00	37.78	40.24	50.85	33.70	19.29	33.63	16.34	34.54
47	53.02	43.92	46.48	39.29	41.45	43.61	52.23	23.69	23.81	31.48	39.90
48	34.87	34.58	40.47	43.50	31.61	48.36	57.74	18.22	21.51	28.31	35.92
49	37.42	49.72	40.97	42.04	34.34	37.66	29.81	18.13	19.15	27.80	33.71
50	59.13	30.48	42.19	31.28	36.35	33.98	36.93	19.85	28.82	27.16	34.62

Continued on next page

Table S1 – continued from previous page

chr	length1	length2	length3	length4	length5	length6	length7	length8	length9	length10	mean_length
51	37.90	32.72	41.77	55.95	35.59	36.83	37.02	18.72	25.46	26.86	34.88
52	42.35	40.63	30.80	41.75	29.23	61.97	47.46	24.50	20.90	24.18	36.38
53	31.49	33.48	44.13	42.65	44.78	40.13	37.28	19.71	21.35	22.35	33.73
54	44.57	42.40	46.78	25.49	25.36	35.66	41.79	19.04	19.75	21.74	32.26
55	48.35	51.93	57.05	39.83	26.60	58.97	46.87	23.30	23.57	29.67	40.61
56	36.09	53.91	36.14	43.36	25.17	39.63	70.83	22.65	22.82	22.14	37.27
57	37.69	36.87	40.15	55.89	26.88	41.51	46.47	17.09	23.83	17.40	34.38
58	38.37	34.27	55.39	35.49	25.71	50.51	43.81	21.76	18.17	21.41	34.49
59	58.91	28.51	48.07	25.27	26.89	36.02	45.81	20.14	15.69	27.87	33.32
60	34.30	34.14	37.60	31.69	22.72	41.45	45.54	24.77	16.28	27.99	31.65
61	21.50	34.36	43.28	34.06	26.90	43.08	47.90	32.47	19.65	30.71	33.39
62	39.93	15.28	50.23	35.46	30.80	61.49	49.28	21.99	20.88	22.47	34.78
63	38.87	29.43	38.02	31.53	25.58	42.17	56.46	21.00	29.78	22.49	33.53
64	49.99	26.51	80.86	28.37	19.27	49.31	46.77	17.95	21.83	44.79	38.57
65	34.89	29.23	38.61	30.61	29.72	46.53	38.83	18.55	20.09	27.00	31.41
66	27.14	37.80	42.82	34.96	31.27	38.58	35.87	18.96	8.64	19.80	29.58
67	42.29	37.81	56.30	33.97	36.60	36.89	45.62	19.87	10.24	25.51	34.51
68	39.21	28.53	48.94	31.22	28.94	34.36	40.97	24.16	10.16	15.16	30.17
69	29.35	28.30	44.03	39.61	33.63	30.94	45.23	24.89	12.37	20.79	30.91
70	29.11	25.76	34.80	36.91	26.24	31.16	54.68	21.62	13.02	22.43	29.57
71	41.65	26.95	27.85	29.93	38.19	30.35	37.51	22.16	14.60	30.37	29.96
72	33.68	26.48	33.69	23.02	22.98	32.89	38.37	29.08	16.09	32.03	28.83
73	40.98	26.12	42.36	26.50	33.54	41.35	44.97	22.26	19.25	15.93	31.33
74	36.08	41.67	39.16	30.16	29.69	18.44	49.49	17.35	16.88	12.50	29.14
75	40.70	32.38	29.08	27.16	28.79	30.17	38.47	25.29	11.79	12.48	27.63
76	23.73	32.81	24.13	28.14	29.87	30.35	47.44	15.98	12.05	14.02	25.85
77	33.87	25.95	38.77	31.57	19.55	25.41	47.56	15.16	11.98	13.50	26.33
78	37.12	22.09	39.47	27.31	17.66	26.56	41.49	8.30	16.91	9.87	24.68
79	29.56	21.56	17.38	34.71	23.06	19.48	42.37	14.49	14.71	16.84	23.42
80	35.26	34.65	40.52	31.51	24.52	25.54	36.40	15.11	22.40	18.67	28.46
81	24.78	24.16	29.76	35.27	23.05	35.53	34.20	12.45	16.40	11.55	24.72
82	34.62	24.67	24.59	25.30	30.26	44.37	27.41	15.64	18.96	12.68	25.85
83	32.62	30.02	18.72	23.96	25.48	23.39	42.51	18.38	22.82	12.26	25.02
84	33.29	21.69	17.24	24.07	24.85	30.81	42.05	11.77	17.43	17.36	24.05
85	31.17	27.20	32.11	29.23	29.93	24.94	22.02	11.90	14.71	18.47	24.17
86	27.24	26.58	23.35	41.77	28.04	31.07	27.41	8.54	18.45	15.01	24.74
87	29.71	15.62	24.66	34.07	23.01	31.72	45.25	8.05	19.35	18.47	24.99
88	27.97	25.89	23.08	35.91	24.82	32.86	33.17	13.38	6.73	14.15	23.80
89	39.20	29.65	22.76	27.64	30.16	26.22	34.41	10.74	14.68	16.56	25.20
90	34.27	34.20	15.60	30.80	35.19	25.93	41.98	12.51	15.79	19.38	26.56
91	30.36	36.09	31.87	36.38	26.48	24.47	39.74	12.65	17.65	11.74	26.74
92	33.85	22.13	19.49	35.94	35.33	32.98	15.98	12.36	12.23	13.11	23.34
93	35.75	30.06	29.67	34.50	25.34	31.04	49.03	14.04	16.09	13.69	27.92
94	31.62	31.06	34.66	28.48	21.22	25.74	32.98	14.26	17.38	13.36	25.08
95	31.90	36.58	13.53	30.08	40.71	27.39	36.83	9.66	17.48	16.92	26.11
96	29.97	36.66	44.04	30.13	23.88	32.30	30.50	12.32	15.47	10.08	26.53
97	34.93	31.04	22.48	39.57	35.25	24.23	45.97	9.96	9.79	15.50	26.87
98	20.84	27.08	29.51	27.04	37.78	33.50	38.57	8.82	23.57	15.04	26.18
99	36.43	29.84	26.73	36.75	26.43	30.16	28.65	13.56	14.26	14.02	25.68
100	33.16	47.32	28.44	35.25	28.14	28.27	29.79	11.96	12.84	16.81	27.20

Table S2. *Carassius auratus i* values were dissected from each metaphase and added into separate table. The last column (*mean_i*) shows arithmetic mean of the *i* value used for dot plot (Figure 1).

chr	i1	i2	i3	i4	i5	i6	i7	i8	i9	i10	mean_i
1	48.70	47.88	47.80	47.52	47.50	48.09	47.22	49.41	49.07	49.02	48.22
2	48.03	47.86	47.75	47.46	47.40	46.06	47.05	49.00	48.52	48.80	47.79
3	47.42	46.54	46.70	47.14	47.27	45.77	46.91	48.69	47.56	48.11	47.21
4	47.07	45.29	46.46	45.78	47.06	45.26	46.72	48.06	47.13	47.59	46.64
5	45.47	43.38	45.77	44.99	46.75	44.78	46.16	46.46	46.15	47.39	45.73
6	44.81	42.66	44.92	43.89	46.35	44.57	45.84	45.74	45.75	47.36	45.19
7	44.78	42.55	44.84	43.86	45.32	43.96	44.92	45.49	45.21	46.77	44.77
8	44.30	42.54	43.77	43.43	45.06	43.56	44.78	45.07	45.21	46.08	44.38
9	43.49	41.79	42.99	41.80	44.93	43.23	44.36	43.85	44.81	45.85	43.71
10	43.15	40.89	42.16	41.14	44.93	43.14	43.42	43.63	44.78	45.73	43.30
11	42.28	40.51	42.08	40.92	44.54	42.93	42.32	42.88	44.71	45.72	42.89
12	40.95	39.99	41.57	40.23	44.38	42.87	42.05	42.54	44.53	44.90	42.40
13	40.64	39.13	40.97	39.98	44.23	42.25	41.20	42.44	44.12	44.80	41.98
14	39.79	38.75	40.13	39.69	42.09	42.07	40.24	42.26	44.06	44.21	41.33
15	39.70	38.73	38.90	39.63	42.05	42.04	39.87	42.04	43.52	44.09	41.06
16	39.07	38.66	38.57	39.62	41.81	41.89	39.80	42.00	43.44	43.99	40.89
17	36.68	36.62	37.48	39.55	41.08	41.61	39.50	41.76	43.19	43.59	40.10
18	36.44	36.41	36.71	39.34	40.70	41.12	39.47	41.68	42.44	43.55	39.79
19	36.24	36.38	36.49	37.60	40.37	40.91	39.17	41.19	42.21	43.44	39.40
20	36.09	36.29	36.48	36.59	40.17	40.33	38.55	41.00	41.93	43.28	39.07
21	36.09	36.26	36.17	36.43	40.03	40.26	38.53	40.98	41.87	42.68	38.93
22	35.67	36.19	36.00	36.42	38.96	39.54	38.23	40.91	41.31	42.58	38.58
23	35.54	35.71	35.99	36.26	38.80	39.08	37.81	40.61	40.38	42.52	38.27
24	35.38	35.52	35.69	36.20	37.49	38.91	37.40	40.53	40.09	42.46	37.97
25	35.25	35.10	35.63	36.05	36.67	38.78	37.03	40.31	39.67	41.80	37.63
26	35.11	34.81	35.16	35.93	35.91	38.56	36.87	40.13	39.36	41.59	37.34
27	34.78	34.81	34.79	35.72	35.80	38.43	36.75	39.69	39.27	41.44	37.15
28	34.61	34.79	34.77	35.59	35.48	38.06	36.57	39.64	39.09	41.39	37.00
29	34.54	34.33	34.56	35.55	35.18	37.35	36.49	39.42	39.01	41.37	36.78
30	34.30	34.08	34.47	35.48	35.14	36.72	36.20	39.18	38.77	41.14	36.55
31	34.27	34.04	34.38	35.40	34.84	36.41	36.09	39.15	38.69	40.86	36.41
32	34.14	34.01	34.22	35.26	34.77	36.41	36.01	39.06	38.68	38.52	36.11
33	33.96	34.00	34.22	35.16	34.76	36.37	35.83	38.77	38.52	38.29	35.99
34	33.92	33.65	34.11	35.02	34.60	36.37	35.80	38.50	36.76	37.73	35.65
35	33.91	33.38	34.02	34.99	33.92	36.27	35.75	38.23	36.64	37.56	35.47
36	33.89	33.34	33.98	34.67	33.82	36.08	35.44	38.20	36.47	36.85	35.27
37	33.69	33.05	33.88	34.55	33.73	35.94	35.20	36.67	36.45	36.61	34.98
38	33.59	32.65	33.59	34.43	33.39	35.80	35.19	36.31	35.89	36.15	34.70
39	33.46	32.48	33.28	34.35	33.08	35.67	34.75	36.14	35.85	36.01	34.51
40	32.52	32.46	33.22	33.00	33.03	35.64	34.30	36.07	35.60	35.87	34.17
41	32.51	32.42	32.93	32.45	32.59	35.59	34.08	36.07	34.92	35.80	33.94
42	32.47	32.25	32.93	32.27	31.76	35.46	33.97	35.61	34.81	35.78	33.73
43	32.17	32.19	32.92	32.27	31.65	35.41	33.39	35.48	34.59	35.70	33.58
44	32.14	32.17	32.73	31.63	27.36	35.26	33.15	35.47	34.54	35.60	33.00
45	32.08	31.52	32.67	31.13	26.74	35.22	33.14	35.37	34.02	35.52	32.74
46	31.90	31.35	32.39	30.99	25.37	35.18	32.89	35.30	33.95	35.40	32.47
47	31.68	30.86	32.15	27.83	23.46	34.98	32.86	35.21	33.78	35.00	31.78
48	31.47	30.51	31.50	27.10	0.00	34.73	31.74	35.07	33.30	34.89	29.03
49	31.42	29.72	31.42	26.61	0.00	34.68	31.38	34.94	33.22	34.85	28.82
50	31.40	29.68	31.39	24.72	0.00	34.64	31.27	34.58	33.15	34.58	28.54

Continued on next page

Table S2 – continued from previous page

chr	i1	i2	i3	i4	i5	i6	i7	i8	i9	i10	mean_i
51	31.19	29.13	30.94	24.51	0.00	34.15	31.16	34.56	32.64	34.57	28.29
52	30.99	29.09	30.94	24.10	0.00	34.13	30.91	33.79	32.45	34.46	28.09
53	30.57	28.85	30.84	20.77	0.00	33.98	30.82	33.61	32.06	34.42	27.59
54	30.46	28.80	30.33	0.00	0.00	33.73	30.46	33.59	31.76	34.40	25.35
55	29.58	28.74	30.08	0.00	0.00	33.34	30.27	33.56	31.34	34.33	25.12
56	28.84	28.63	29.92	0.00	0.00	33.23	30.13	33.55	31.18	34.25	24.97
57	28.80	27.62	29.72	0.00	0.00	32.39	30.04	33.39	31.03	34.24	24.72
58	28.09	27.15	29.34	0.00	0.00	32.17	29.78	33.37	30.85	33.58	24.43
59	27.93	26.48	28.90	0.00	0.00	30.90	29.50	33.37	30.79	33.34	24.12
60	27.21	24.27	28.79	0.00	0.00	30.84	29.02	33.30	29.35	33.15	23.59
61	26.52	24.03	27.75	0.00	0.00	30.40	28.64	33.04	29.22	33.11	23.27
62	21.77	0.00	26.37	0.00	0.00	30.28	28.59	32.89	28.99	33.06	20.19
63	20.90	0.00	26.33	0.00	0.00	29.95	28.39	32.73	28.53	32.94	19.98
64	0.00	0.00	26.30	0.00	0.00	28.73	28.21	32.35	27.42	32.85	17.59
65	0.00	0.00	25.43	0.00	0.00	28.36	27.79	32.15	23.92	32.18	16.98
66	0.00	0.00	24.90	0.00	0.00	27.73	27.35	31.75	0.00	31.84	14.36
67	0.00	0.00	24.07	0.00	0.00	22.84	27.04	31.70	0.00	31.80	13.74
68	0.00	0.00	23.08	0.00	0.00	22.39	26.24	30.68	0.00	31.10	13.35
69	0.00	0.00	20.91	0.00	0.00	0.00	26.06	30.30	0.00	30.31	10.76
70	0.00	0.00	15.52	0.00	0.00	0.00	25.84	29.79	0.00	30.08	10.12
71	0.00	0.00	0.00	0.00	0.00	0.00	24.72	26.60	0.00	29.50	8.08
72	0.00	0.00	0.00	0.00	0.00	0.00	24.52	25.89	0.00	26.60	7.70
73	0.00	0.00	0.00	0.00	0.00	0.00	24.51	25.76	0.00	21.15	7.14
74	0.00	0.00	0.00	0.00	0.00	0.00	24.44	21.34	0.00	0.00	4.58
75	0.00	0.00	0.00	0.00	0.00	0.00	24.34	14.65	0.00	0.00	3.90
76	0.00	0.00	0.00	0.00	0.00	0.00	23.45	0.00	0.00	0.00	2.34
77	0.00	0.00	0.00	0.00	0.00	0.00	22.92	0.00	0.00	0.00	2.29
78	0.00	0.00	0.00	0.00	0.00	0.00	20.79	0.00	0.00	0.00	2.08
79	0.00	0.00	0.00	0.00	0.00	0.00	17.36	0.00	0.00	0.00	1.74
80	0.00	0.00	0.00	0.00	0.00	0.00	17.28	0.00	0.00	0.00	1.73
81	0.00	0.00	0.00	0.00	0.00	0.00	7.08	0.00	0.00	0.00	0.71
82	0.00	0.00	0.00	0.00	0.00	0.00	0.00	0.00	0.00	0.00	0.00
83	0.00	0.00	0.00	0.00	0.00	0.00	0.00	0.00	0.00	0.00	0.00
84	0.00	0.00	0.00	0.00	0.00	0.00	0.00	0.00	0.00	0.00	0.00
85	0.00	0.00	0.00	0.00	0.00	0.00	0.00	0.00	0.00	0.00	0.00
86	0.00	0.00	0.00	0.00	0.00	0.00	0.00	0.00	0.00	0.00	0.00
87	0.00	0.00	0.00	0.00	0.00	0.00	0.00	0.00	0.00	0.00	0.00
88	0.00	0.00	0.00	0.00	0.00	0.00	0.00	0.00	0.00	0.00	0.00
89	0.00	0.00	0.00	0.00	0.00	0.00	0.00	0.00	0.00	0.00	0.00
90	0.00	0.00	0.00	0.00	0.00	0.00	0.00	0.00	0.00	0.00	0.00
91	0.00	0.00	0.00	0.00	0.00	0.00	0.00	0.00	0.00	0.00	0.00
92	0.00	0.00	0.00	0.00	0.00	0.00	0.00	0.00	0.00	0.00	0.00
93	0.00	0.00	0.00	0.00	0.00	0.00	0.00	0.00	0.00	0.00	0.00
94	0.00	0.00	0.00	0.00	0.00	0.00	0.00	0.00	0.00	0.00	0.00
95	0.00	0.00	0.00	0.00	0.00	0.00	0.00	0.00	0.00	0.00	0.00
96	0.00	0.00	0.00	0.00	0.00	0.00	0.00	0.00	0.00	0.00	0.00
97	0.00	0.00	0.00	0.00	0.00	0.00	0.00	0.00	0.00	0.00	0.00
98	0.00	0.00	0.00	0.00	0.00	0.00	0.00	0.00	0.00	0.00	0.00
99	0.00	0.00	0.00	0.00	0.00	0.00	0.00	0.00	0.00	0.00	0.00
100	0.00	0.00	0.00	0.00	0.00	0.00	0.00	0.00	0.00	0.00	0.00
